# Identification of Conserved Cross-Reactive B-Cell Epitopes in CPV1 and CPV2 L1 Proteins with Vaccine Potential

**DOI:** 10.3390/vaccines14060512

**Published:** 2026-06-06

**Authors:** Yuge Wang, Yingyi Chen, Kaixin Wang, Youqing Yuan, Haojie Sun, Youming Yuan, Jixian Wang, Zhicai Yang, Yi Yang, Naidong Wang, Deyong Duan, Aibing Wang

**Affiliations:** 1 Laboratory of Animal Disease Prevention and Control and Animal Model, Hunan Provincial Key Laboratory of Protein Engineering in Animal Vaccines, College of Veterinary Medicine, Hunan Agricultural University (HUNAU), Changsha 410128, China; yuge.wang@stu.hunau.edu.cn (Y.W.); 15773134998@163.com (Y.C.); wangkaixin0708@163.com (K.W.); shj2661139643@stu.hunau.edu.cn (H.S.); wjx612@stu.hunau.edu.cn (J.W.); yzc.ly.26@stu.hunau.edu.cn (Z.Y.); yiyang@hunau.edu.cn (Y.Y.); naidongwang@hunau.edu.cn (N.W.); 2 Department of Chemistry, University College London, London WC1H 0AJ, UK; youqing.yuan.23@ucl.ac.uk; 3 College of Food Science & Nutritional Engineering, China Agricultural University, Beijing 100083, China; yuanyouming06@outlook.com

**Keywords:** canine papillomavirus, L1 protein, antigenic epitope, immunogenicity, vaccine development

## Abstract

**Background/Objectives:** Canine papillomavirus (CPV) is an important viral pathogen associated with papillomatosis in dogs, with canine papillomavirus type 1 (CPV1) and type 2 (CPV2) among the most prevalent and clinically relevant genotypes. The L1 capsid protein is a major immunogenic antigen of papillomaviruses; however, conserved linear B-cell epitopes shared between CPV genotypes remain poorly defined. This study aimed to identify conserved cross-reactive B-cell epitopes within CPV1 and CPV2 L1 proteins and to evaluate their preliminary immunoreactivity. **Methods:** Conserved linear B-cell epitopes were predicted through integrated bioinformatic and structural analyses based on sequence conservation and surface accessibility. Three candidate epitopes were selected. Recombinant CPV1 and CPV2 L1 proteins were expressed in *Escherichia coli* (*E. coli*), purified, used as recombinant L1 antigens, together with BSA-conjugated synthetic epitope peptides for mouse immunization. Antigen-specific IgG responses were assessed by ELISA, antigen-associated IFN-γ responses were evaluated by ELISpot, and cross-reactive antibody recognition was assessed by Western blot. **Results:** Recombinant L1 proteins induced strong antigen-specific IgG responses in mice. The selected peptides induced detectable but weaker humoral responses compared with the recombinant L1 proteins. Among the three epitopes, TPSGSLV and TVVDNTR elicited antibodies that recognized both CPV1 and CPV2 L1 proteins, while the epitope VIVPKVS showed minimal or no detectable immunoreactivity. ELISpot analysis showed only modest antigen-associated IFN-γ responses, particularly in peptide-immunized groups. **Conclusions:** This study identified conserved cross-reactive linear B-cell epitope candidates within CPV1 and CPV2 L1 proteins and provided preliminary immunological evidence supporting their potential relevance for CPV antigen design. However, peptide-induced responses were weaker than those induced by recombinant L1 proteins, and VLP formation, antibody neutralizing activity, and protective efficacy were not evaluated. Further studies in dogs, including optimized antigen-display platforms, neutralization assays, and protection studies, are required to determine the practical value of these epitopes for CPV vaccine development.

## 1. Introduction

Canine papillomavirus (CPV), a member of the *Papillomaviridae* family, is a small, non-enveloped virus with a circular double-stranded DNA genome that primarily infects stratified squamous epithelial cells of canine skin and mucosa [1,2,3]. CPV infection usually causes benign papillomas; however, persistent infection or infection in immunocompromised hosts may contribute to lesion progression and, in rare cases, malignant transformation [2,3,4]. With the continued expansion of the global companion dog population and increasing attention to preventive veterinary medicine, CPV-associated diseases are receiving greater clinical relevance. However, no licensed prophylactic vaccines are currently available for CPV infection, underscoring the need for improved antigen discovery and vaccine-development strategies [2].

Multiple CPV genotypes have been identified, among which CPV1 and CPV2 are the most prevalent and clinically important in dogs [5]. Similarly to other papillomaviruses, CPVs encode the major capsid protein, L1, which constitutes the principal structural component of the viral capsid and is a key target of humoral immune responses. In human papillomavirus (HPV), recombinant L1-based virus-like particles (VLPs) vaccines have shown excellent immunogenicity and durable protective efficacy [6,7,8,9,10,11,12,13,14]. These vaccines mainly rely on conformational epitopes displayed on the native three-dimensional structure of assembled L1 VLPs, which are the major targets of potent neutralizing antibodies. This provides a strong precedent for L1-based papillomavirus vaccine development.

However, the antigenic landscape of CPV L1 proteins remains incompletely characterized, particularly with respect to conserved antigenic regions shared between CPV genotypes. Although conformational epitopes presented by native L1 VLPs are central to protective papillomavirus immunity, conserved linear B-cell epitopes may also have value for cross-reactive antigen design, immunodiagnostic development, and incorporation into optimized multi-epitope or complementary vaccine platforms. Linear epitopes are especially suitable for sequence conservation analysis across related viral genotypes and can be readily adapted for synthetic peptide-based screening and antigen engineering.

Epitope-based vaccine design offers a promising approach for identifying conserved immune targets that may overcome genotype-restricted immune recognition [15,16,17,18,19,20]. By focusing on evolutionarily conserved and predicted surface-accessible regions of viral proteins such as the L1, this approach can support the rational selection of candidate antigenic determinants with potential cross-reactive properties [21,22]. Advances in computational immunology, structural modeling, and epitope prediction algorithms have greatly improved the efficiency and accuracy of identifying such candidates. When integrated with experimental validation, these tools provide a practical framework for defining antigenic regions that may inform future vaccine or diagnostic development [21,23,24,25,26].

In the present study, we aimed to identify conserved linear B-cell epitopes within the L1 proteins of CPV1 and CPV2 through comprehensive bioinformatic and structural analyses. Candidate epitopes were assessed based on predicted antigenicity, sequence conservation, structural accessibility, and non-toxicity. Recombinant L1 proteins expressed in a prokaryotic system were used as recombinant L1 antigens, together with BSA-conjugated synthetic epitope peptides, to evaluate humoral immunoreactivity and cross-reactive antibody recognition in a mouse model. This study identifies conserved cross-reactive B-cell epitope candidates in CPV1 and CPV2 L1 proteins and provides preliminary immunological information that may support future CPV antigen design and vaccine-related studies.

## 2. Materials and Methods

### 2.1. Bioinformatic Prediction and Structural Analysis

The secondary and tertiary structures of CPV1 and CPV2 L1 proteins were analyzed to facilitate antigenic epitope identification. Secondary structural elements, including α-helices, β-strands, and random coils, were predicted using PSIPRED (version 4.0). Linear B-cell epitopes were predicted using ABCpred and Bcepred v2.0. To improve prediction specificity, ABCpred analysis was performed using a threshold of 0.7, which is more stringent than the default threshold of 0.51.

Candidate epitopes were further evaluated for evolutionary conservation using the ConSurf server and screened for potential toxicity using ToxinPred 2.0. Three-dimensional structures of CPV1 and CPV2 L1 proteins were generated using AlphaFold, and predicted epitopes were mapped onto the modeled structures using PyMOL version 2.5 to assess their spatial localization and predicted surface accessibility. Based on integrated analysis of predicted antigenicity, sequence conservation, non-toxicity, and structural accessibility, the selected peptides were used to identify minimal conserved linear B-cell motifs shared between CPV1 and CPV2 L1 proteins, rather than optimized T-cell epitopes or confirmed protective vaccine antigens.

### 2.2. Epitope Peptide Synthesis and BSA Conjugation

Three conserved epitope peptides (VIVPKVS, TPSGSLV, and TVVDNTR) were synthesized by standard solid-phase peptide synthesis by GenScript Biotech, Nanjing, China, with >95% purity. A terminal cysteine residue was added to each peptide during synthesis to enable maleimide-mediated conjugation to bovine serum albumin (BSA), according to the manufacturer’s protocol. The BSA-conjugated peptides were purified by high-performance liquid chromatography (HPLC) and verified by mass spectrometry (MS) before use.

### 2.3. Generation of Recombinant Plasmids

Full-length coding sequences of CPV1 L1 and CPV2 L1 were obtained based on reference sequences of CPV1 (GenBank accession no. D55633) and CPV2 (GenBank accession no. AY722648). The coding sequences were codon-optimized for prokaryotic expression, synthesized, and fused with an N-terminal 6×His tag by Tsingke Biotechnology Co., Ltd., Beijing, China. The optimized CPV1 L1 gene was cloned into the pCold I expression vector, whereas the CPV2 L1 gene was inserted into the pET-28a(+) vector, generating recombinant plasmids pCold I-CPV1 L1 and pET-28a-CPV2 L1, respectively. *Escherichia coli* BL21 (DE3) competent cells were purchased from Beijing Bomaide Gene Technology Co., Ltd., (Beijing, China), and used for recombinant protein expression.

### 2.4. Expression and Purification of Recombinant CPV1 and CPV2 L1 Proteins

Recombinant plasmids pCold I-CPV1 L1 and pET28a-CPV2 L1 were transformed into *E. coli* BL21 (DE3) competent cells. Transformed bacteria were cultured in LB medium at 37 °C until the optical density at 600 nm (OD600) reached 0.6–0.8. CPV1 L1 expression was induced using the pCold system at 16 °C for 24 h, whereas CPV2 L1 expression was induced with 0.5–1 mM IPTG at 25 °C for 10 h. Cells were harvested by centrifugation and lysed by ultrasonic sonication on ice. After clarification by centrifugation, His-tagged L1 proteins were purified using Ni-NTA affinity chromatography, buffer-exchanged into PBS and stored at −80 °C until further use. Purified CPV1 and CPV2 L1 proteins were used as recombinant L1 protein antigens directly in this study in the absence of VLP formation analysis by structural methods such as non-reducing SDS-PAGE, native PAGE, size-exclusion chromatography, dynamic light scattering, or transmission electron microscopy.

### 2.5. SDS-PAGE and Western Blot Analysis of Recombinant Protein Expression

Protein samples were separated by SDS-PAGE on 10% polyacrylamide gels under reducing conditions, alongside a prestained protein molecular weight marker. Proteins were transferred onto PVDF membranes using a semi-dry transfer system at 20 V for 40 min. Membranes were blocked with 5% skim milk in PBST for 1 h at room temperature and incubated overnight at 4 °C with mouse anti-His monoclonal antibody at a 1:5000 dilution. After washing with PBST, membranes were incubated with HRP-conjugated goat anti-mouse IgG secondary antibody at a 1:5000 dilution for 1 h at room temperature. Immunoreactive signals were detected using enhanced chemiluminescence substrate (Thermo Fisher Scientific, Waltham, MA, USA). Protein identity was supported by the expected molecular weight, inducible expression pattern, and anti-His immunoblot reactivity of the purified recombinant proteins.

### 2.6. Animal Immunization and Welfare Monitoring

Female BALB/c mice (6-week-old, *n* = 30) were purchased from Hunan Slack Jingda Laboratory Animal Co., Ltd., (Changsha, Hunan), and acclimated for one week under specific pathogen-free (SPF) conditions. All animal procedures were conducted in accordance with institutional guidelines for the care and use of laboratory animals. Mice were randomly assigned to six groups, with five mice per group: Group 1, recombinant CPV1 L1 protein; Group 2, recombinant CPV2 L1 protein; Group 3, BSA-conjugated peptide 1; Group 4, BSA-conjugated peptide 2; Group 5, BSA-conjugated peptide 3; and Group 6, naïve control.

Purified recombinant CPV1/CPV2 L1 proteins or BSA-conjugated synthetic peptides were emulsified with Freund’s complete adjuvant for primary immunization and Freund’s incomplete adjuvant for booster immunizations. Proteins, 10 μg per mouse, or BSA-conjugated peptides, 50 μg per mouse, were administered subcutaneously in a total volume of 100 μL on day 0, followed by booster immunizations on days 14 and 28. Blood samples were collected on days 14, 28, and 42, and spleens were harvested on day 42 for immunological assays.

Animals were monitored daily after immunization for general health, injection-site reactions, mobility, behavior, and body weight. Humane endpoint criteria included severe local inflammation, impaired mobility, abnormal behavior, marked distress, or body weight loss exceeding 20%.

### 2.7. Sample Collection

#### 2.7.1. Serum Preparation

Blood samples were collected from the retro-orbital plexus under anesthesia. After clotting at room temperature, samples were centrifuged at 800× *g* for 10 min at 4 °C, sera were collected and stored at −20 °C or −80 °C until further analysis.

#### 2.7.2. Splenocyte Isolation

Spleens were aseptically harvested after euthanasia. Single-cell suspensions were prepared by mechanical dissociation through a 200-mesh nylon cell strainer in RPMI-1640 medium. Mononuclear cells were isolated by density gradient centrifugation, washed, counted, and resuspended in RPMI-1640 medium containing 10% fetal bovine serum for subsequent assays.

### 2.8. Immunological Assays

#### 2.8.1. Indirect ELISA

Indirect ELISA was performed to evaluate antigen-specific IgG responses in mouse sera. ELISA plates were coated overnight at 4 °C with purified recombinant CPV1 L1 or CPV2 L1 protein at 5 μg/mL in carbonate buffer, pH 9.6, depending on the immunization group and assay purpose. Sera from mice immunized with recombinant CPV1 L1 or CPV2 L1 were tested against the corresponding homologous recombinant L1 protein. For peptide-immunized groups, plates were coated with recombinant CPV1 and/or CPV2 L1 proteins to determine whether peptide-induced antibodies recognized the recombinant L1 antigens and showed cross-reactive binding.

Plates were blocked with 5% skim milk in PBST and incubated with serially diluted mouse sera at 37 °C for 1 h. After washing, HRP-conjugated goat anti-mouse IgG at a 1:5000 dilution was added and incubated for 1 h at 37 °C. Color development was performed using TMB substrate and terminated with 2 M H_2_SO_4_. Optical density was measured at 450 nm using a microplate reader. Samples were considered ELISA-positive when the OD_450_ value was at least twofold higher than the mean OD450 value of the negative control sera.

#### 2.8.2. IFN-γ ELISpot Assay

IFN-γ ELISpot assays were performed using a precoated IFN-γ ELISpot kit (Dakewe Biotech, Shenzhen, Guangdong, China) according to the manufacturer’s instructions. Splenocytes were seeded at 4 × 10^6^ cells/well in IFN-γ ELISpot plates and stimulated with recombinant CPV1/CPV2 L1 proteins or synthetic peptides at 10 μg/mL. Parallel wells without antigen stimulation were included as background controls. PMA stimulation was used as a positive control.

After incubation, IFN-γ spot-forming cells were developed and counted according to the manufacturer’s instructions. Antigen-specific responses were calculated by subtracting the number of spots in unstimulated wells from those in antigen-stimulated wells. A response was considered positive when spot numbers were above background and at least twofold higher than the corresponding unstimulated control. Given the low number of IFN-γ-secreting cells observed in peptide-stimulated wells, ELISpot data were interpreted cautiously as supportive evidence of antigen-associated cellular responses rather than definitive T-cell epitope activity.

#### 2.8.3. Western Blot Analysis of Antigenicity Reactivity

Purified L1 proteins were separated by SDS-PAGE and transferred onto PVDF membranes. Membranes were blocked with 5% skim milk in PBST for 1 h at room temperature and incubated with mouse anti-L1 or anti-peptide immune sera at a 1:500 dilution, followed by HRP-conjugated goat anti-mouse IgG at a 1:5000 dilution. Immunoreactive signals were detected using an enhanced chemiluminescence substrate. Weak bands comparable to those observed in negative-control serum lanes were interpreted as background or non-specific signals and were not considered definitive antigen-specific reactivity. Full Western blot images can be found in File S1.

### 2.9. Statistical Analysis

**Figure 5:**
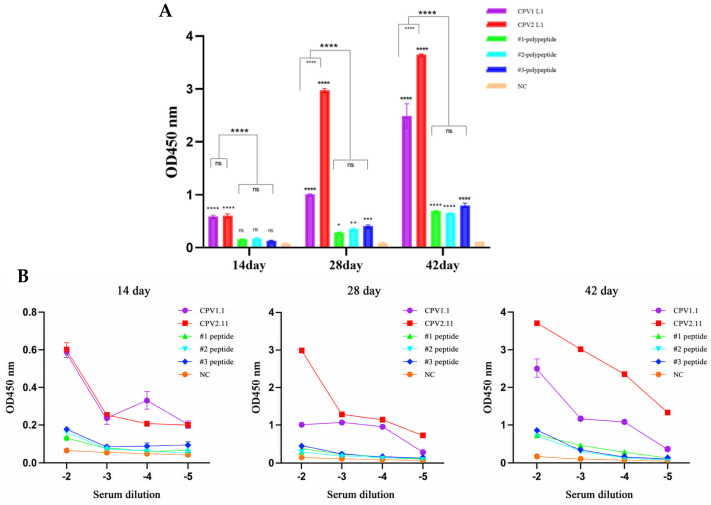
Humoral immune responses induced by CPV1 and CPV2 L1 proteins and peptide immunization. Indirect ELISA was performed to evaluate antigen-specific IgG responses in sera from immunized mice. Plates were coated with purified recombinant CPV1 L1 or CPV2 L1 protein, as indicated. (**A**) Time course of antigen-specific IgG titers in serum collected at 14, 28, and 42 days after primary immunization. (**B**) Antigen-specific IgG levels measured in serially diluted sera (10^−2^–10^−7^). Data are presented as mean ± SD (*n* = 5 mice per group). Statistical significance among groups was analyzed using the Kruskal–Wallis test followed by Dunn’s multiple-comparisons test. A *p* value < 0.05 was considered statistically significant. Statistical significance is indicated as **** *p* < 0.0001, *** *p* < 0.001, ** *p* < 0.01, * *p* < 0.05 and ns, not significant (*p* > 0.05).

All experiments were performed with at least three independent biological replicates. Quantitative data are presented as the mean ± standard deviation (SD). Statistical analyses were conducted using GraphPad Prism software (version 9.0, GraphPad Software, Boston, MA, USA). Normality was assessed using the Shapiro–Wilk test, and homogeneity of variance was evaluated using Levene’s test before parametric analysis. For multiple-group comparisons, one-way ANOVA followed by Tukey’s post hoc test was used when assumptions of normality and equal variance were met; otherwise, the Kruskal–Wallis test followed by Dunn’s multiple-comparisons test was applied. For datasets with relatively small sample sizes, including Figure 5, the Kruskal–Wallis test followed by Dunn’s multiple-comparisons test was used. Comparisons between two groups were performed using Student’s *t*-test when appropriate. A *p* value < 0.05 was considered statistically significant.

## 3. Results

### 3.1. Structural Prediction and Modeling of CPV1 and CPV2 L1 Proteins

**Figure 1:**
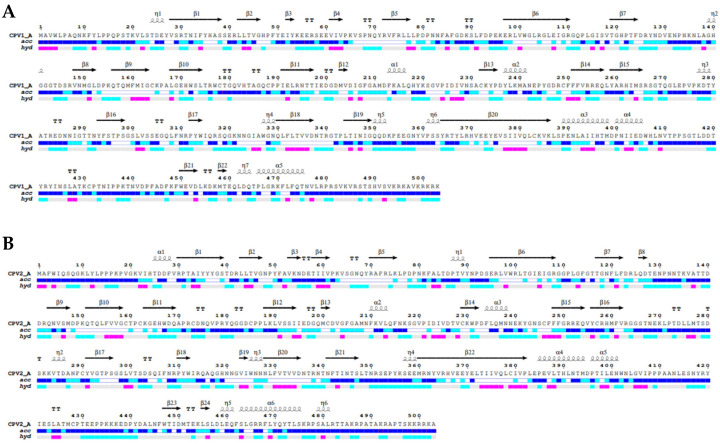

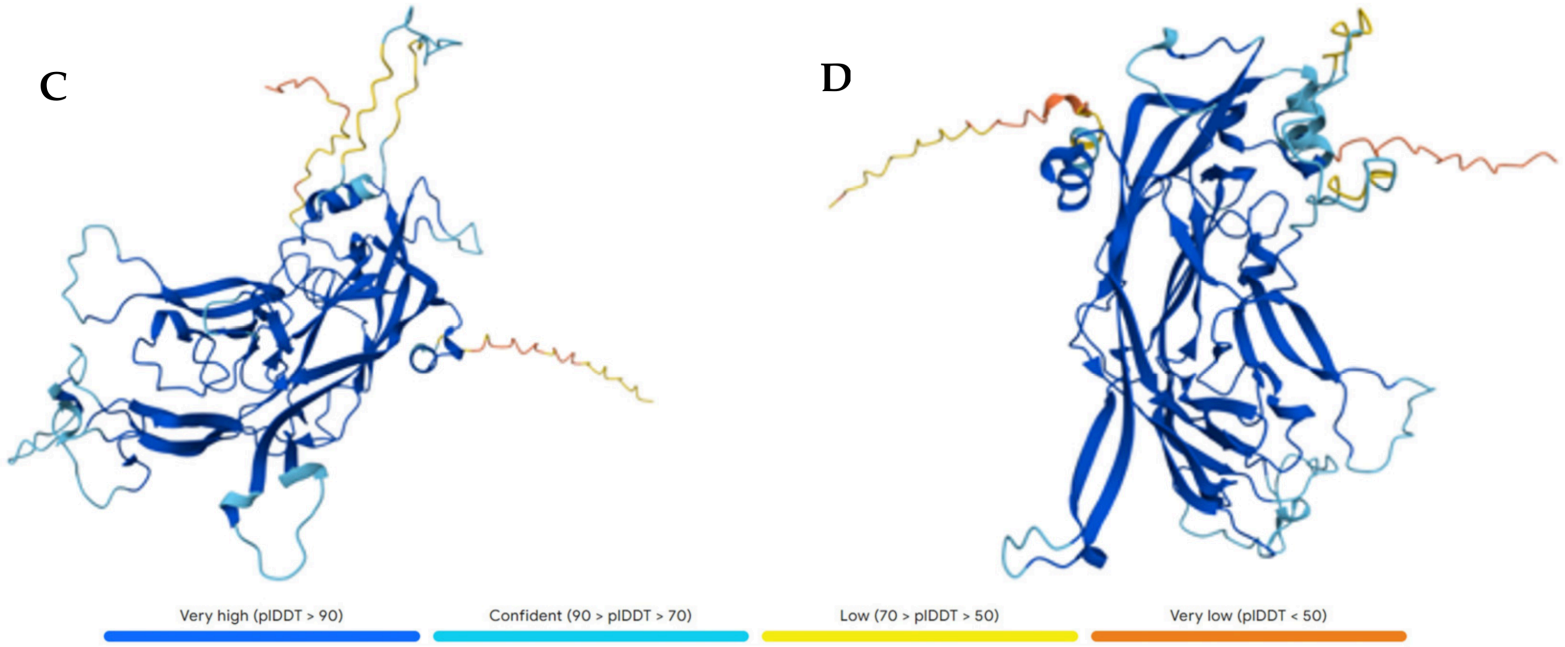
Secondary and tertiary structure predictions of CPV1 and CPV2 L1 proteins. (**A**) Predicted secondary structure of the CPV1 L1 protein; (**B**) predicted secondary structure of the CPV2 L1 protein. In the hydrophilicity plots, blue regions indicate hydrophilic residues, whereas red regions indicate hydrophobic residues. (**C**) Predicted tertiary structure of the CPV1 L1 protein; (**D**) predicted tertiary structure of the CPV2 L1 protein. The color gradient represents the predicted local distance difference test (pLDDT) confidence scores: dark blue (>90), light blue (70–90), orange (50–70), and yellow (<50).

The secondary structures of CPV1 and CPV2 L1 proteins were predicted using established bioinformatics tools. Both proteins exhibited a β-strand-rich architecture with interspersed α-helices and coil regions, consistent with the general structural organization of papillomavirus L1 proteins (Figure 1A,B). Analysis of surface accessibility and hydrophilicity indicated that multiple hydrophilic regions were predicted to be surface-exposed, whereas hydrophobic residues were more frequently located in internal regions. These features suggest that the CPV1 and CPV2 L1 proteins contain potentially accessible antigenic regions suitable for further epitope prediction.

The tertiary structures of CPV1 and CPV2 L1 proteins were predicted using AlphaFold3. The resulting models showed conserved core domains with relatively high confidence scores, whereas several loop regions and terminal regions showed lower confidence scores, consistent with their predicted structural flexibility (Figure 1C,D). Because the structural models were computational predictions, they were used primarily to support epitope localization and predicted surface accessibility, rather than to infer native capsid or VLP assembly.

### 3.2. Prediction of Potential Linear B-Cell Epitopes

Linear B-cell epitopes of CPV1 and CPV2 L1 proteins were predicted using ABCpred and Bcepred, with a threshold of 0.7 applied in ABCpred to improve prediction stringency. Candidate epitopes were further screened using ToxinPred to exclude potentially toxic sequences and were evaluated based on predicted antigenicity, conservation, and structural accessibility. Through this integrated analysis, several potential linear B-cell epitopes were identified in CPV1 and CPV2 L1 proteins (Table 1). Among them, three conserved or highly similar epitope regions shared by CPV1 and CPV2 were selected for further experimental evaluation: VIVPKVS, TPSGSLV, and TVVDNTR. These short peptides were chosen as minimal conserved linear motifs for cross-reactive B-cell epitope screening, rather than as optimized T-cell epitopes or complete vaccine antigens.

### 3.3. Conservation Analysis of Predicted Epitopes

**Figure 2:**
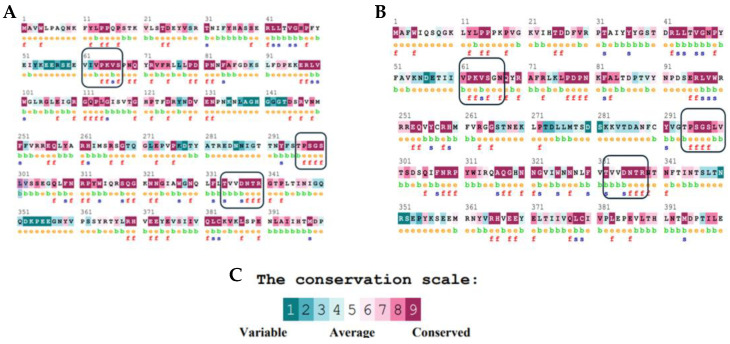
Conservation analysis of CPV1 and CPV2 L1 proteins. (**A**) Conservation profile of the CPV1 L1 protein; (**B**) Conservation profile of the CPV2 L1 protein; (**C**) Conservation score scale derived from ConSurf analysis. In the ConSurf annotation, residues labeled “e” indicate exposed residues, “b” indicate buried residues, “f” denote highly conserved functional residues exposed on the protein surface, and “s” denote highly conserved residues located within the protein core.

To further evaluate the evolutionary conservation, residue conservation within the L1 proteins was analyzed using the ConSurf server v2.0. The selected epitope regions contained conserved residues, some of which were predicted to be exposed or located near surface-accessible regions (Figure 2). The epitope VIVPKVS contained multiple conserved residues, whereas TPSGSLV and TVVDNTR also showed conserved features across CPV1 and CPV2 L1 proteins. These results supported their selection as conserved linear B-cell epitope candidates for experimental evaluation. However, because conservation and predicted accessibility do not necessarily indicate neutralizing or protective activity, these epitopes were assessed only for preliminary immunoreactivity and cross-reactive antibody recognition.

### 3.4. Structural Mapping of Candidate Epitopes

**Figure 3:**
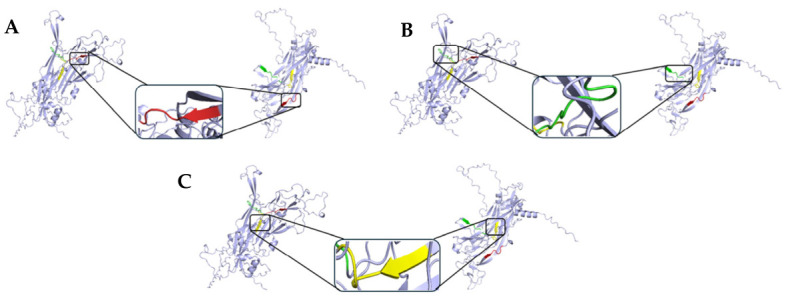
Structural localization of selected B-cell epitopes on the CPV L1 protein. (**A**) Epitope 1 (VIVPKVS) is shown in red; (**B**) epitope 2 (TPSGSLV) is shown in green; (**C**) epitope 3 (TVVDNTR) is shown in yellow.

The three selected epitopes were mapped onto the predicted CPV L1 structures using PyMOL. The epitopes VIVPKVS, TPSGSLV, and TVVDNTR were mainly located in the surface-exposed or transitional regions of the L1 protein structure (Figure 3), suggesting that they may be accessible for antibody recognition and supporting their evaluation as candidate linear B-cell epitopes. Because this analysis was based on predicted protein structures, the structural mapping should be interpreted as supportive bioinformatic evidence rather than direct experimental structural validation.

### 3.5. Expression and Purification of Recombinant CPV1 and CPV2 L1 Proteins

**Figure 4:**
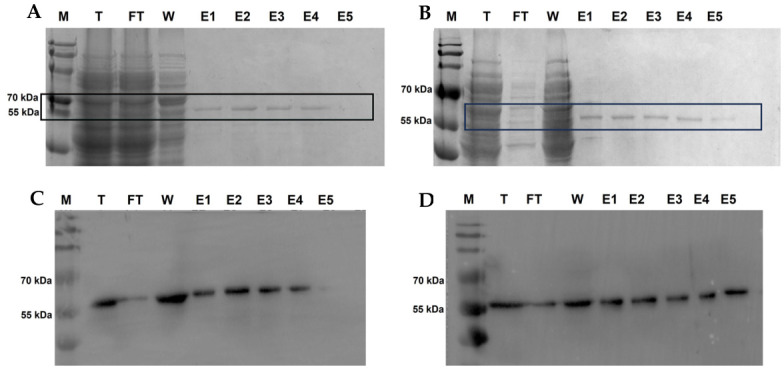
Prokaryotic expression, purification, and identification of CPV1 and CPV2 L1 proteins. (**A**,**B**) SDS-PAGE analysis of recombinant CPV1 L1 (**A**) and CPV2 L1 (**B**) proteins expressed in *E. coli* and purified by Ni-NTA affinity chromatography. (**C**,**D**) Western blot analysis of CPV1 L1 (**C**) and CPV2 L1 (**D**) proteins using an anti-His tag primary antibody and HRP-conjugated goat anti-mouse IgG secondary antibody. M, protein molecular weight marker (8–195 kDa); T, total protein; FT, flow-through; W, wash fraction; E1–E5, elution fractions. The target protein bands (~55–60 kDa) are indicated in (**A**,**B**).

To generate recombinant L1 antigens for immunization and immunological assays, CPV1 and CPV2 L1 genes were cloned into prokaryotic expression vectors and expressed in *Escherichia coli*. After induction, the recombinant proteins were purified using Ni-NTA affinity chromatography. SDS-PAGE analysis showed enriched protein bands at approximately 55–60 kDa in the elution fractions, consistent with the expected molecular weights of recombinant CPV1 and CPV2 L1 proteins (Figure 4A,B). Western blot analysis using an anti-His antibody further detected immunoreactive bands at the expected molecular weight range in the purified protein fractions (Figure 4C,D), supporting successful expression and purification of both recombinant CPV1 and CPV2 L1 proteins. These proteins were used as recombinant L1 antigens in this study; their native oligomeric state was not determined, and VLP assembly was not experimentally confirmed.

### 3.6. Humoral Immune Responses Induced by CPV L1 Proteins and Epitope Peptides

Antigen-specific IgG responses induced by recombinant CPV1 L1 and CPV2 L1, and BSA-conjugated epitope peptides were evaluated by indirect ELISA on days 14, 28, and 42 after primary immunization. ELISA plates were coated with recombinant CPV1 and/or CPV2 L1 proteins according to the immunization group and assay purpose. Samples were considered ELISA-positive when the OD450 value was at least twofold higher than the mean OD450 value of the negative control sera. As shown in Figure 5A, recombinant CPV1 and CPV2 L1 proteins induced clear antigen-specific IgG responses that increased over time, whereas the negative control group maintained low baseline levels throughout the experiment. Compared with the recombinant L1 protein-immunized groups, the peptide-immunized groups showed detectable but substantially weaker antibody responses. At day 14, some anti-peptide serum responses were close to the positivity threshold, and were therefore interpreted cautiously.

To further assess antibody titration profiles, sera were serially diluted and analyzed by ELISA (Figure 5B). The repeated ELISA titration assay showed a clearer dilution-dependent decrease in OD450 values for anti-protein sera and selected anti-peptide sera. Recombinant L1 protein-immunized sera retained stronger reactivity across serial dilutions, while peptide-induced reactivity was weaker and declined more rapidly. Signals at very high dilutions that approached the negative-control background were interpreted cautiously and were not considered evidence of strong antibody reactivity.

Together, these results indicate that recombinant CPV1 and CPV2 L1 proteins induced stronger humoral responses than the selected short linear peptides, while selected peptide antigens induced detectable but comparatively modest antibody responses in mice.

### 3.7. Cellular Immune Responses

**Figure 6:**
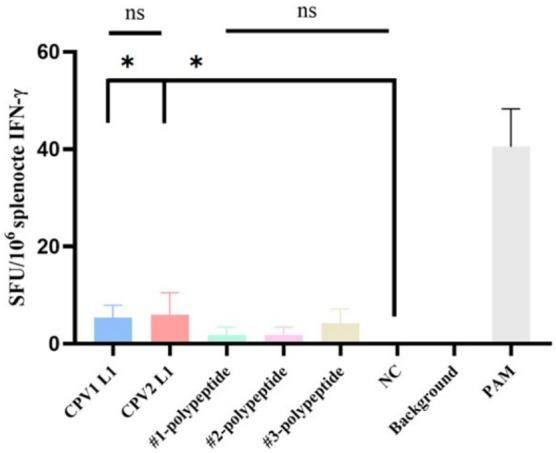
ELISpot analysis of IFN-γ responses in mouse splenocytes stimulated with CPV1/CPV2 L1 proteins and epitope peptides. Mouse splenocytes were stimulated with recombinant CPV1 L1 protein, CPV2 L1 protein, and epitope peptides (#1–#3), respectively. Negative control (NC) and background control were included, and phytohemagglutinin (PMA) was used as a positive control. IFN-γ-producing cells were quantified as spot-forming units (SFU) per 10^6^ splenocytes. Data are presented as mean ± SD (*n* = 5 mice per group). Statistical significance is indicated as * *p* < 0.05, and ns, not significant (*p* > 0.05).

Splenocytes isolated on day 42 primary immunization were stimulated in vitro with recombinant CPV1 and CPV2 L1 proteins or epitope peptides, and IFN-γ secretion were assessed by ELISpot. Parallel unstimulated wells were included as background controls, and antigen-specific responses were calculated after background subtraction. The positive control induced robust IFN-γ spot formation, confirming the functional responsiveness of the isolated splenocytes (Figure 6). Recombinant L1 protein-stimulated groups showed detectable IFN-γ responses, whereas peptide-stimulated groups showed low IFN-γ spot numbers that were not statistically significant compared with the negative control group. Therefore, although peptide-immunized groups showed detectable IFN-γ spot formation, the current data do not provide sufficient evidence that the tested short peptides directly induced robust T-cell activation. These results are best interpreted as limited or modest antigen-associated cellular responses under the experimental conditions used.

### 3.8. Antigenic Reactivity and Cross-Reactive Recognition of CPV1/CPV2 L1 Proteins

**Figure 7:**
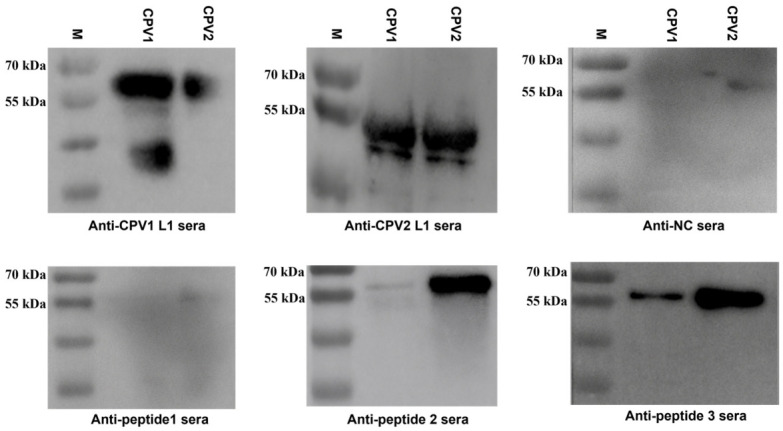
Western blot analysis of the immunoreactivity of mouse sera against purified CPV1 and CPV2 L1 proteins. Purified CPV1 and CPV2 L1 proteins were used as antigens to evaluate the immunoreactivity of sera from mice immunized with CPV1 L1 protein, CPV2 L1 protein, or epitope peptides #1–#3. Mouse sera were used as primary antibodies, followed by HRP-conjugated goat anti-mouse IgG as the secondary antibody. Sera from mice immunized with CPV1 or CPV2 L1 proteins exhibited strong reactivity with both L1 proteins, with clear bands detected at approximately 60 kDa, indicating cross-reactive antibody responses. Sera from peptide #2- and #3-immunized mice also exhibited cross-reactivity with both CPV1 and CPV2 L1 proteins, whereas serum from the peptide #1-immunized group showed no detectable reactivity. No specific bands were observed in the negative control (NC) group, confirming the absence of non-specific binding.

Western blot analysis was performed to determine whether antibodies induced by recombinant L1 proteins or selected peptides could recognize CPV1 and CPV2 L1 antigens. Purified recombinant CPV1 and CPV2 L1 proteins were used as target antigens, and immune sera from vaccinated mice were used as primary antibodies. Sera from mice immunized with recombinant CPV1 or CPV2 L1 proteins showed strong reactivity with recombinant L1 antigens, with bands detected at approximately 55–60 kDa (Figure 7), indicating that recombinant L1 protein immunization induced antibodies capable of recognizing L1 antigens. Among peptide-immunized groups, sera against peptide 2 (TPSGSLV) and peptide 3 (TVVDNTR) showed detectable recognition of both CPV1 and CPV2 L1 proteins, suggesting cross-reactive antibody binding, whereas peptide 1 (VIVPKVS) serum showed minimal or no clear immunoreactivity. A faint signal observed in one CPV2 lane probed with peptide 1 serum was comparable to the weak signal in the negative-control lane and was therefore interpreted as likely background or non-specific reactivity rather than definitive peptide-specific recognition. Only bands clearly stronger than the negative-control background were considered specific antigen-reactive signals. These results suggest that TPSGSLV and TVVDNTR are conserved linear B-cell epitope candidates with cross-reactive antigen-binding potential, although neutralizing activity and protective efficacy remain to be determined.

## 4. Discussion

CPVs are increasingly recognized as a clinically relevant pathogens in companion animals [2,3,4,5,27,28]. Although CPV infection is often associated with benign papillomas, persistent infection, immunosuppression, and genotype diversity may complicate disease control. The absence of licensed prophylactic CPV vaccines highlights the need to better define conserved antigenic regions that may support future vaccine and diagnostic development. Among the identified CPV genotypes, CPV1 and CPV2 are among the most prevalent and clinically important in dogs [3,5,29], making them appropriate targets for comparative antigenic analysis.

Papillomavirus L1 is the major capsid protein and a central target of humoral immune responses. In HPV, licensed L1-based VLP vaccines provide strong protection primarily by inducing neutralizing antibodies against conformational epitopes displayed on the native three-dimensional VLP surface [9,11,14,30,31,32,33,34]. Therefore, conformational epitopes and assembled VLP structures remain the most established basis for protective papillomavirus vaccination. The continued clinical importance of HPV-associated diseases also supports ongoing refinement of papillomavirus vaccine strategies [35,36,37,38]. The present study does not challenge this principle, rather, it focuses on conserved linear B-cell epitopes within CPV1 and CPV2 L1 proteins as candidate antigenic motifs that may be useful for cross-reactive antigen design, immunodiagnostic development, or incorporation into future optimized multi-epitope or display-based platforms [39,40,41,42,43,44,45]. Using integrated bioinformatic and structural analyses, we identified three conserved or highly similar linear motifs, VIVPKVS, TPSGSLV, and TVVDNTR, within CPV1 and CPV2 L1 proteins. These epitopes were selected based on predicted antigenicity, sequence conservation, non-toxicity, and predicted structural accessibility. Structural mapping suggested that the selected motifs were located in surface-accessible or transitional regions of the predicted L1 structures, supporting their potential availability for antibody recognition. However, because these structural data were derived from computational models, they should be interpreted as supportive evidence rather than direct experimental proof of native epitope exposure.

Recombinant CPV1 and CPV2 L1 proteins expressed in *E. coli* induced stronger antibody responses than the short synthetic peptides. This finding is expected, as full-length L1 proteins contain multiple linear and conformational antigenic regions and provide broader immune stimulation than short peptides. In contrast, the selected 7-mer peptides represent minimal conserved linear motifs. Such short peptides may have limited intrinsic immunogenicity and are unlikely to fully reflect naturally processed or conformationally presented epitopes [33,34,42]. Therefore, the peptide-induced antibody responses should be interpreted as preliminary evidence of immunoreactivity rather than evidence that these peptides are effective standalone immunogens.

Among the three tested peptides, TPSGSLV and TVVDNTR induced sera that recognized both recombinant CPV1 and CPV2 L1 proteins by Western blot, suggesting cross-reactive antigen binding. In contrast, VIVPKVS showed minimal or no clear immunoreactivity. These findings indicate that TPSGSLV and TVVDNTR may represent truly conserved cross-reactive linear B-cell epitope candidates. However, antibody binding detected by ELISA or Western blot does not necessarily indicate neutralizing activity. Because live-virus or pseudovirus neutralization assays were not performed, the functional relevance of the peptide-induced antibodies remains undetermined.

The ELISpot results should also be interpreted cautiously. Although detectable IFN-γ spot formation was observed under some stimulation conditions, the overall number of IFN-γ-secreting cells was low, particularly in peptide-stimulated groups. In addition, peptide-immunized groups did not show statistically significant increases compared with the negative control. Therefore, these data do not provide strong evidence that the tested short peptides directly induced robust T-cell activation. The ELISpot findings are better considered limited, supportive observations of antigen-associated cellular responses. More rigorous evaluation of CPV1/CPV2 L1-specific T-cell immunity will require optimized peptide pools, longer peptide constructs, helper epitopes, intracellular cytokine staining, T-cell proliferation assays, and flow cytometry-based phenotyping [42,43,44,45].

The recombinant CPV1 and CPV2 L1 proteins used in this study were expressed in a prokaryotic system and used as recombinant L1 protein antigens. Although papillomavirus L1 proteins have intrinsic self-assembly potential, bacterial expression does not necessarily ensure complete VLP formation [30,32,34]. In this study, direct structural analyses, such as non-reducing SDS-PAGE, native PAGE, size-exclusion chromatography, dynamic light scattering, or transmission electron microscopy, were not performed. Therefore, the recombinant L1 proteins should not be described as confirmed VLPs. Future structural characterization will be needed to determine whether these proteins form monomers, capsomeres, higher-order oligomers, or VLP-like particles.

Despite these limitations, the identification of conserved cross-reactive linear B-cell epitope candidates may still have value for future antigen design. Minimal linear motifs such as TPSGSLV and TVVDNTR are unlikely to function effectively as standalone vaccine antigens, but they may be useful when incorporated into larger optimized constructs. Potential strategies include multi-epitope antigens, carrier-assisted formulations, recombinant fusion proteins, nanoparticle display, VLP-display systems, or nucleic acid-based platforms such as DNA or mRNA constructs [35,36,37,39,40,41,42,43,44,45,46,47,48]. These approaches may enhance antigen density, improve B-cell activation, and better integrate humoral and cellular immune stimulation.

Several limitations should be emphasized. First, immunogenicity was evaluated in mice rather than in dogs, the natural host of CPV infection. Second, peptide-induced antibody responses were weaker and less consistent than those induced by recombinant L1 proteins. Third, neutralizing activity and protective efficacy were not assessed. Fourth, VLP assembly or native oligomeric status of recombinant L1 proteins was not experimentally determined. Finally, the short peptides used in this study were designed to identify minimal conserved linear B-cell motifs, not optimized vaccine-ready antigens or T-cell epitopes.

In summary, this study identifies conserved cross-reactive linear B-cell epitope candidates within CPV1 and CPV2 L1 proteins and provides preliminary immunological evidence supporting their antigenic relevance. The findings may inform future CPV antigen design and provide candidate motifs for optimized multi-epitope, carrier-based, or display-platform strategies [35,36,37,39,40,41,42,43,44,45,46,47,48]. However, further studies in the canine host, including structural characterization, neutralization assays, optimized antigen-delivery systems, and protective-efficacy evaluation, are required to determine their practical value for CPV vaccine development.

## 5. Conclusions

In conclusion, this study identified conserved cross-reactive linear B-cell epitope candidates within the L1 proteins of CPV1 and CPV2. Among the three selected peptides, TPSGSLV and TVVDNTR showed detectable cross-reactive antibody recognition of recombinant CPV1 and CPV2 L1 proteins, whereas VIVPKVS showed minimal or no clear immunoreactivity. Recombinant L1 proteins induced stronger humoral responses than the short synthetic peptides, indicating that these minimal linear epitopes are unlikely to function effectively as standalone immunogens.

The present findings provide preliminary immunological evidence and candidate epitope information that may support future CPV antigen design. However, the peptide-induced responses were weaker than those elicited by recombinant L1 proteins, and the neutralizing activity, protective efficacy, native oligomeric status, and VLP formation of the recombinant L1 proteins were not evaluated in this study. Further studies in dogs, including optimized antigen-display systems, structural characterization, neutralization assays, and protection experiments, will be required to determine the practical value of these epitopes for CPV vaccine development.

## 6. Patent

The authors declare that a patent application has been filed covering aspects of the conserved epitopes and their potential applications in vaccine development described in this study.

## Figures and Tables

**Table 1:** Prediction of candidate epitopes for potential B-cell dominance shared by both CPV1 and 2 L1 protein.

Genotype	Epitope Sequence	Start Position (aa)	ABCpred Score	Toxicity
CPV1	VIVPKVS	61	0.88	Non-Toxic
	NDVENPN	128	0.71	
	RHIMSRSGT	261	0.84	
	TYATRED	279	0.93	
	TPSGSLV	296	0.74	
	TVVDNTRG	334	0.75	
	RKAVKRKRK	495	0.72	
CPV2	IIVPKVS	59	0.85	Non-Toxic
	DTENPNN	127	0.89	
	KVATTDDR	135	0.9	
	HMFVRGGSTN	259	0.8	
	TPSGSLV	294	0.83	
	TVVDNTRN	332	0.8	
	CPTEEPPKKKE	428	0.87	

## Data Availability

All data supporting the findings of this study are included within the article. Additional raw data are available from the corresponding author upon reasonable request.

## Supplementary Materials

The following supporting information can be downloaded at: https://www.mdpi.com/article/10.3390/vaccines14060512/s1, File S1: Full Western blot images.

## Abbreviations

The following abbreviations are used in this manuscript:

CPV1	Canine Papillomavirus Types 1
CPV2	Canine Papillomavirus Types 2

## References

[1] Zhou D., Luff J., Paul S., Alkhilaiwi F., Usuda Y., Wang N., Affolter V., Moore P., Schlegel R., Yuan H. (2015). Complete Genome Sequence of Canine Papillomavirus Virus Type 12. Genome Announc..

[2] Feng Y., Wang K., Zhou D., Yuan Y., Chen Y., Wang J., Sun H., Huang X., Peng X., Yang Y. (2025). Canine papillomavirus: Status of diagnostic methods and vaccine innovations. Virol. J..

[3] Munday J.S., Knight C.G. (2024). Papillomaviruses and Papillomaviral Disease in Dogs and Cats: A Comprehensive Review. Pathogens.

[4] Chang C.-Y., Chen W.-T., Haga T., Yamashita N., Lee C.-F., Tsuzuki M., Chang H.-W. (2020). The Detection and Association of Canine Papillomavirus with Benign and Malignant Skin Lesions in Dogs. Viruses.

[5] Lange C., Jennings S., Diallo A., Lyons J. (2019). Canine papillomavirus types 1 and 2 in classical papillomas: High abundance, different morphological associations and frequent co-infections. Vet. J..

[6] Williamson A.-L. (2023). Recent Developments in Human Papillomavirus (HPV) Vaccinology. Viruses.

[7] Naupu P.N., van Zyl A.R., Rybicki E.P., Hitzeroth I.I. (2020). Immunogenicity of Plant-Produced Human Papillomavirus (HPV) Virus-Like Particles (VLPs). Vaccines.

[8] Garbuglia A.R., Lapa D., Sias C., Capobianchi M.R., Del Porto P. (2020). The Use of Both Therapeutic and Prophylactic Vaccines in the Therapy of Papillomavirus Disease. Front. Immunol..

[9] Orozco J.J., Carter J.J., Koutsky L.A., Galloway D.A. (2005). Humoral Immune Response Recognizes a Complex Set of Epitopes on Human Papillomavirus Type 6 L1 Capsomers. J. Virol..

[10] Buck C.B., Day P.M., Trus B.L. (2013). The papillomavirus major capsid protein L1. Virology.

[11] Bishop B., Dasgupta J., Klein M., Garcea R.L., Christensen N.D., Zhao R., Chen X.S. (2007). Crystal Structures of Four Types of Human Papillomavirus L1 Capsid Proteins. J. Biol. Chem..

[12] Schiller J.T., Lowy D.R. (2012). Understanding and learning from the success of prophylactic human papillomavirus vaccines. Nat. Rev. Microbiol..

[13] Markowitz L.E., Schiller J.T. (2021). Human Papillomavirus Vaccines. J. Infect. Dis..

[14] Zhang X., Li S., Modis Y., Li Z., Zhang J., Xia N., Zhao Q. (2015). Functional assessment and structural basis of antibody binding to human papillomavirus capsid. Rev. Med. Virol..

[15] Caradonna T.M., Schmidt A.G. (2021). Protein engineering strategies for rational immunogen design. npj Vaccines.

[16] Shawan M.M.A.K., Sharma A.R., Halder S.K., Arian T.A., Shuvo M.N., Sarker S.R., Hasan M.A. (2023). Advances in Computational and Bioinformatics Tools and Databases for Designing and Developing a Multi-Epitope-Based Peptide Vaccine. Int. J. Pept. Res. Ther..

[17] Mahmoudvand S., Ghaleh H.E.G., Jalilian F.A., Farzanehpour M., Dorostkar R. (2023). Design of a multi-epitope-based vaccine consisted of immunodominant epitopes of structural proteins of SARS-CoV-2 using immunoinformatics approach. Biotechnol. Appl. Biochem..

[18] Maleki A., Russo G., Palumbo G.A.P., Pappalardo F. (2022). In silico design of recombinant multi-epitope vaccine against influenza A virus. BMC Bioinform..

[19] Ciazynska K. (2024). Turning vaccine design on its head. Nat. Struct. Mol. Biol..

[20] Akbari E., Seyedinkhorasani M., Bolhassani A. (2023). Conserved multiepitope vaccine constructs: A potent HIV-1 therapeutic vaccine in clinical trials. Braz. J. Infect. Dis..

[21] Wei Y., Qiu T., Ai Y., Zhang Y., Xie J., Zhang D., Luo X., Sun X., Wang X., Qiu J. (2024). Advances of computational methods enhance the development of multi-epitope vaccines. Brief. Bioinform..

[22] Kharisma V.D., Ansori A.N.M., Jakhmola V., Rizky W.C., Widyananda M.H., Probojati R.T., Murtadlo A.A.A., Rebezov M., Scherbakov P., Burkov P. (2022). Multi-Strain Human Papillomavirus (HPV) Vaccine Innovation via Computational Study: A Mini Review. Res. J. Pharm. Technol..

[23] Villanueva-Flores F., Sanchez-Villamil J.I., Garcia-Atutxa I. (2025). AI-driven epitope prediction: A systematic review, comparative analysis, and practical guide for vaccine development. npj Vaccines.

[24] Geyer M., Peterlin B. (2001). Domain assembly, surface accessibility and sequence conservation in full length HIV-1 Nef. FEBS Lett..

[25] Bravi B. (2024). Development and use of machine learning algorithms in vaccine target selection. npj Vaccines.

[26] Ananya, Panchariya D.C., Karthic A., Singh S.P., Mani A., Chawade A., Kushwaha S. (2024). Vaccine design and development: Exploring the interface with computational biology and AI. Int. Rev. Immunol..

[27] Bernard H.-U., Burk R.D., Chen Z., Van Doorslaer K., zur Hausen H., de Villiers E.-M. (2010). Classification of papillomaviruses (PVs) based on 189 PV types and proposal of taxonomic amendments. Virology.

[28] Munday J.S., Kiupel M. (2009). Papillomavirus-Associated Cutaneous Neoplasia in Mammals. Vet. Pathol..

[29] de Alcântara B.K., Alfieri A.A., Rodrigues W.B., Otonel R.A., Lunardi M., Headley S.A. (2014). Identification of canine papillomavirus type 1 (CPV1) DNA in dogs with cutaneous papillomatosis. Pesqui. Vet. Bras..

[30] Christensen N.D., Hopfl R., DiAngelo S.L., Cladel N.M., Patrick S.D., Welsh P.A., Budgeon L.R., Reed C.A., Kreider J.W. (1994). Assembled baculovirus-expressed human papillomavirus type 11 L1 capsid protein virus-like particles are recognized by neutralizing monoclonal antibodies and induce high titres of neutralizing antibodies. J. Gen. Virol..

[31] Stanley M. (2009). Immune responses to human papilloma viruses. Indian J. Med. Res..

[32] Buck C.B., Pastrana D.V., Lowy D.R., Schiller J.T. (2004). Efficient intracellular assembly of papillomaviral vectors. J. Virol..

[33] Pedroza-Saavedra A., Rodriguez-Ocampo A.N., Salazar-Piña A., Perez-Morales A.C., Chihu-Amparan L., Maldonado-Gama M., Cruz-Valdez A., Esquivel-Guadarrama F., Gutierrez-Xicotencatl L. (2021). Differential Antibody Response against Conformational and Linear Epitopes of the L1 Proteins from Human Papillomavirus Types 16/18 Is Observed in Vaccinated Women or with Uterine Cervical Lesions. Vaccines.

[34] Hines J., Ghim S., Christensen N., Kreider J., Barnes W., Schlegel R., Jenson A. (1994). Role of conformational epitopes expressed by human papillomavirus major capsid proteins in the serologic detection of infection and prophylactic vaccination. Gynecol. Oncol..

[35] Purcell A.W., McCluskey J., Rossjohn J. (2007). More than one reason to rethink the use of peptides in vaccine design. Nat. Rev. Drug Discov..

[36] Bulla A.C.S., da Silva A.S., Sereno B.P., Dias M.F.R., da Silva M.L. (2025). Computational Methods in Immunoinformatics: Epitope Discovery and Diagnostic Applications. ACS Omega.

[37] Castro K.M., Scheck A., Xiao S., Correia B.E. (2022). Computational design of vaccine immunogens. Curr. Opin. Biotechnol..

[38] Kheirvari M., Liu H., Tumban E. (2023). Virus-like Particle Vaccines and Platforms for Vaccine Development. Viruses.

[39] Stanley M., Pinto L.A., Trimble C. (2012). Human papillomavirus vaccines—Immune responses. Vaccine.

[40] Schiller J.T., Müller M. (2015). Next generation prophylactic human papillomavirus vaccines. Lancet Oncol..

[41] Roden R.B.S., Stern P.L. (2018). Opportunities and challenges for human papillomavirus vaccination in cancer. Nat. Rev. Cancer.

[42] Tumban E. (2019). A Current Update on Human Papillomavirus-Associated Head and Neck Cancers. Viruses.

[43] Cates Z.P., Facciuolo A., Scruten E., Kusalik A., Napper S. (2025). Peptide immunoarrays for rationale development of vaccines with enhanced cross-reactivity. PLoS ONE.

[44] Liu J., Zhang Z., Pu W., Pan X., Li P., Bai Q., Liang S., Li C., Yu Y., Yao H. (2024). A multi-epitope subunit vaccine providing broad cross-protection against diverse serotypes of Streptococcus suis. npj Vaccines.

[45] Doorbar J. (2005). The papillomavirus life cycle. J. Clin. Virol..

[46] Rigo M.M., Fasoulis R., Conev A., Hall-Swan S., Antunes D.A., Kavraki L.E. (2022). SARS-Arena: Sequence and Structure-Guided Selection of Conserved Peptides from SARS-related Coronaviruses for Novel Vaccine Development. Front. Immunol..

[47] Xu J., Sekiguchi T., Boonyakida J., Kato T., Park E.Y. (2023). Display of multiple proteins on engineered canine parvovirus-like particles expressed in cultured silkworm cells and silkworm larvae. Front. Bioeng. Biotechnol..

[48] Zhou G., Luo Z., Zhang Z., Cao S., Li Y. (2025). MS2 virus-like particles as a versatile platform for multi-disease vaccines: A review. Front. Immunol..

