# Supplementary figures and images for: Identification of Conserved Cross-Reactive B-Cell Epitopes in CPV1 and CPV2 L1 Proteins with Vaccine Potential
Source: Vaccines (Basel). 2026 Jun 6;14(6):512. doi: 10.3390/vaccines14060512 (PMC13307585; doi:10.3390/vaccines14060512)

## Slide 1
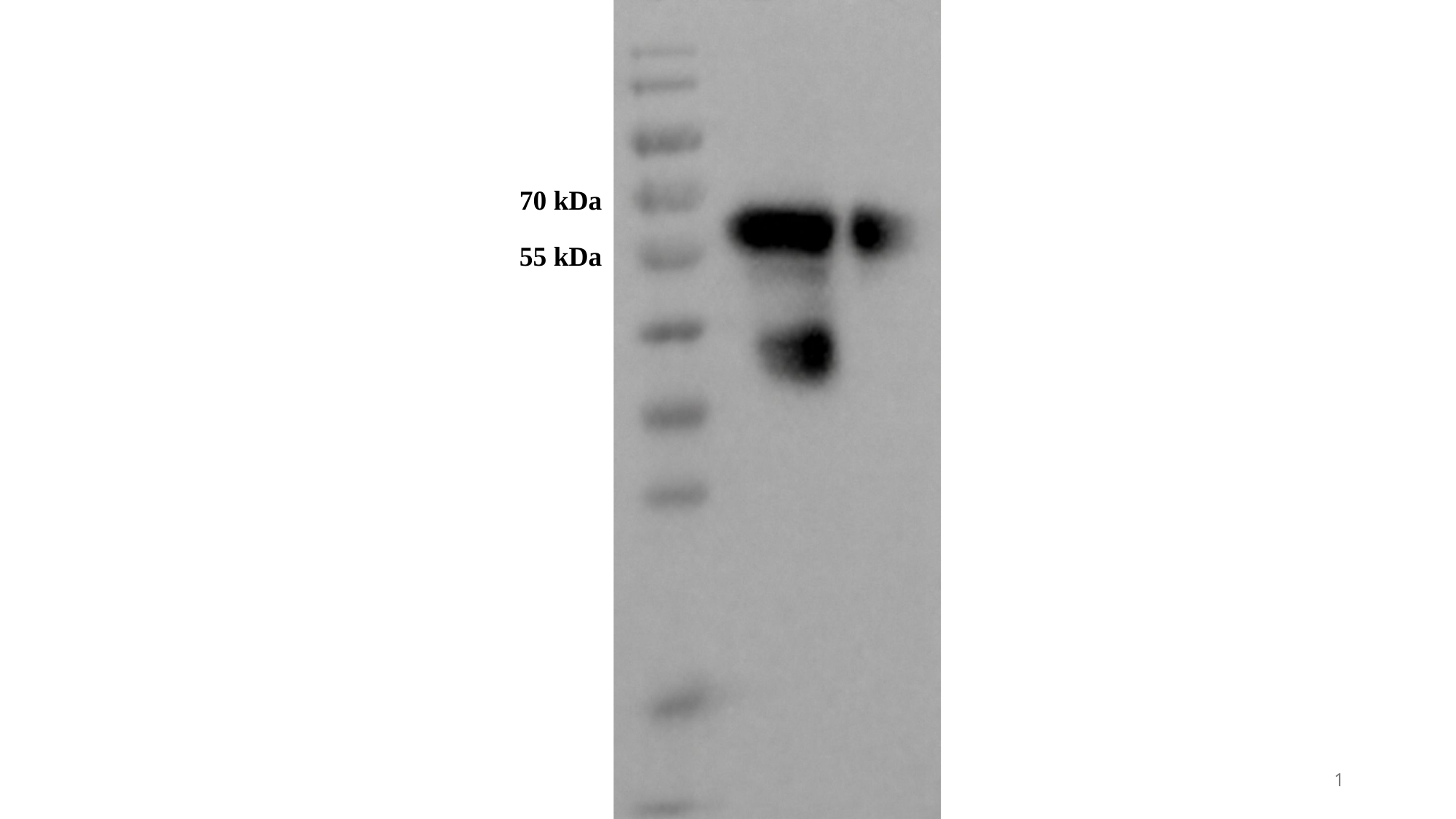

70 kDa
55 kDa
1

## Slide 2
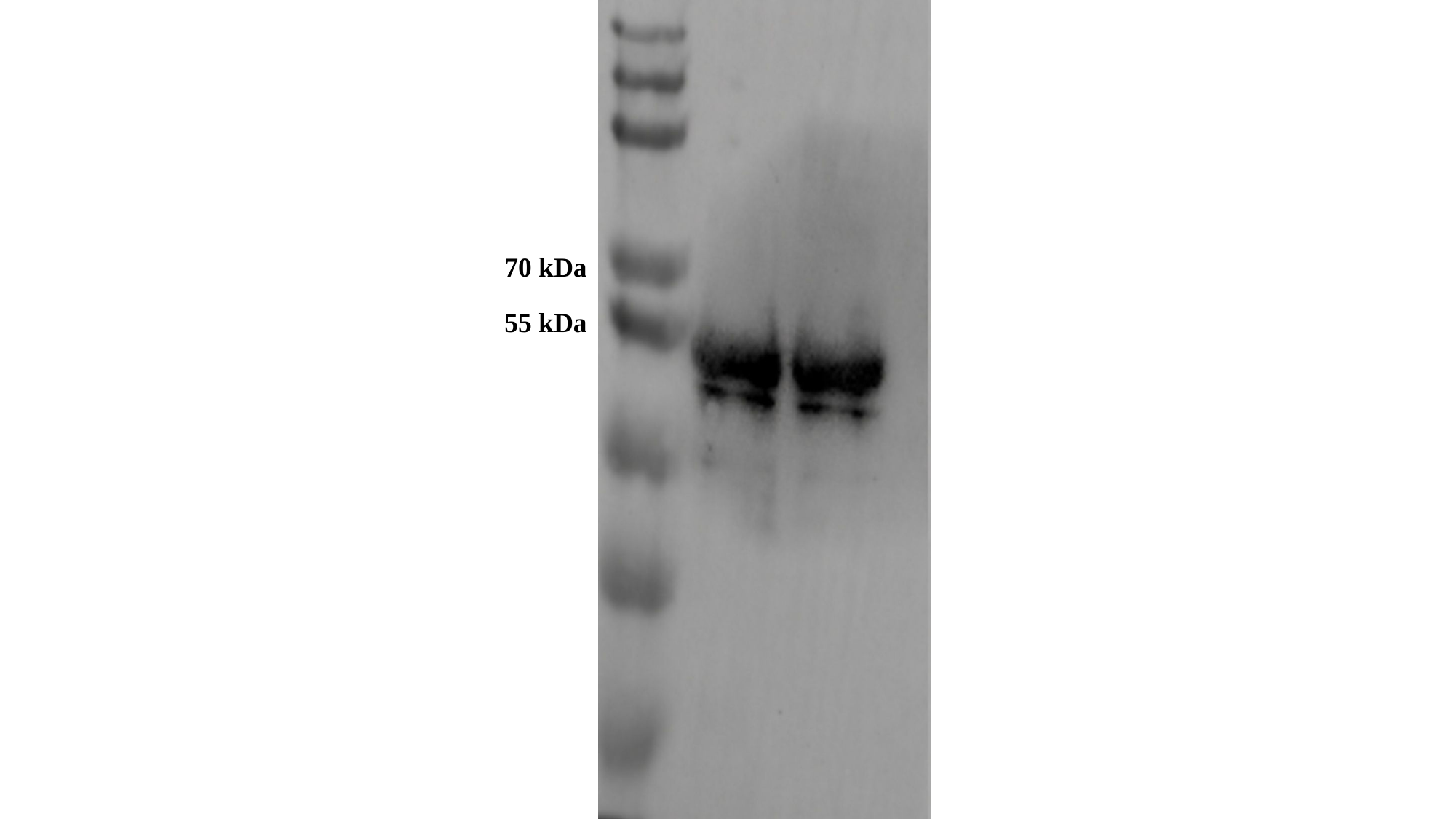

70 kDa
55 kDa

## Slide 3
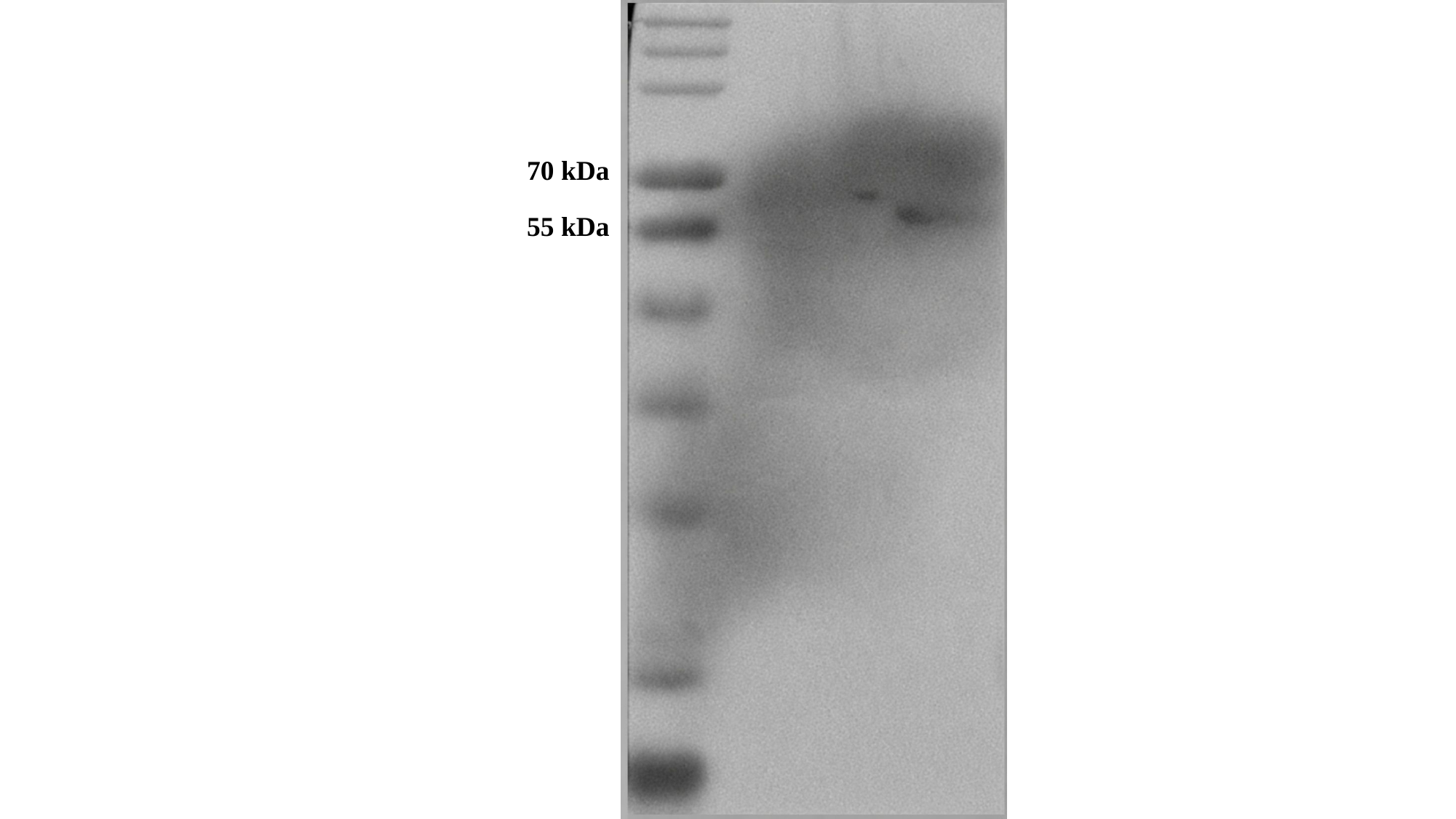

70 kDa
55 kDa

## Slide 4
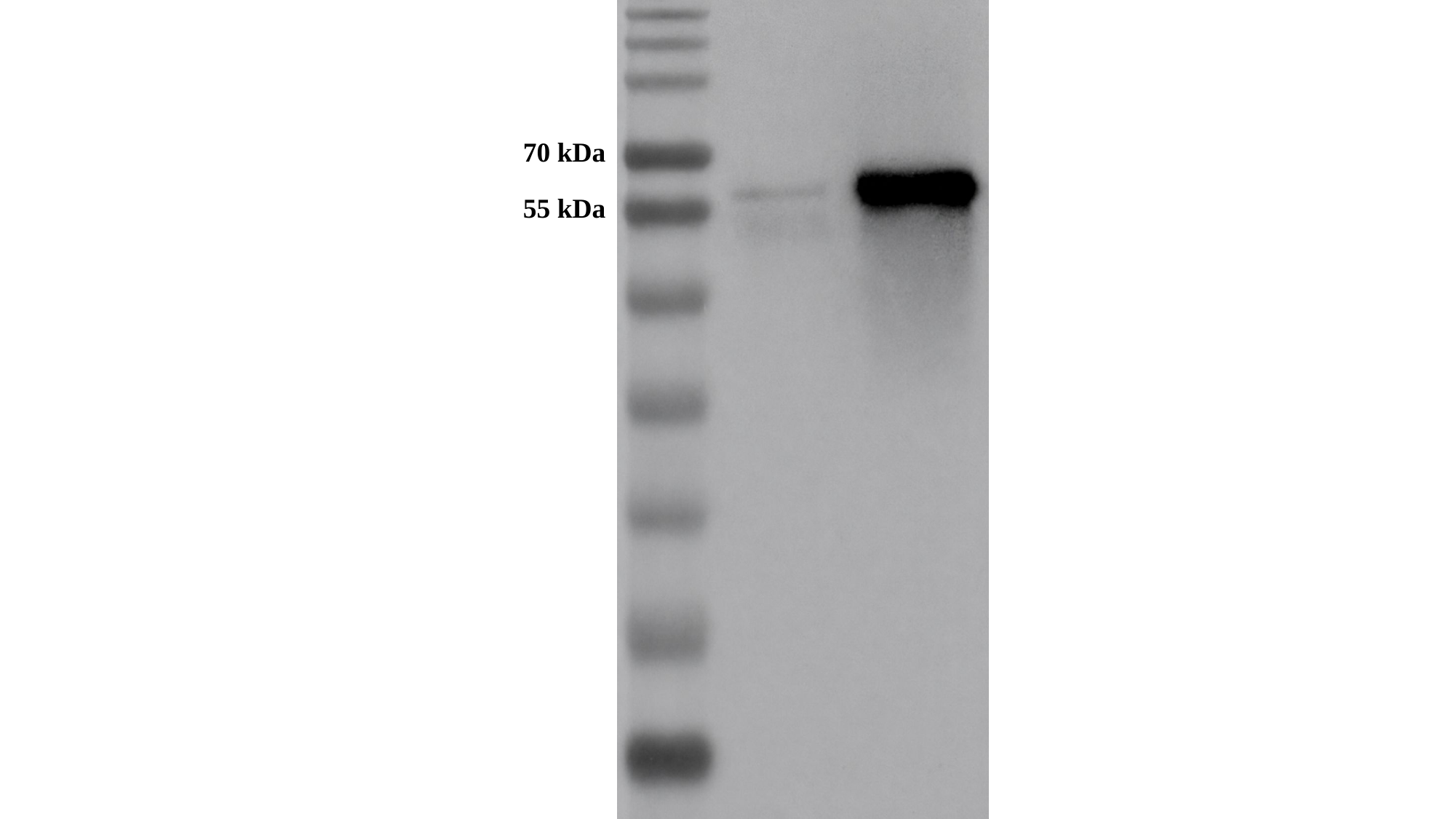

70 kDa
55 kDa

## Slide 5
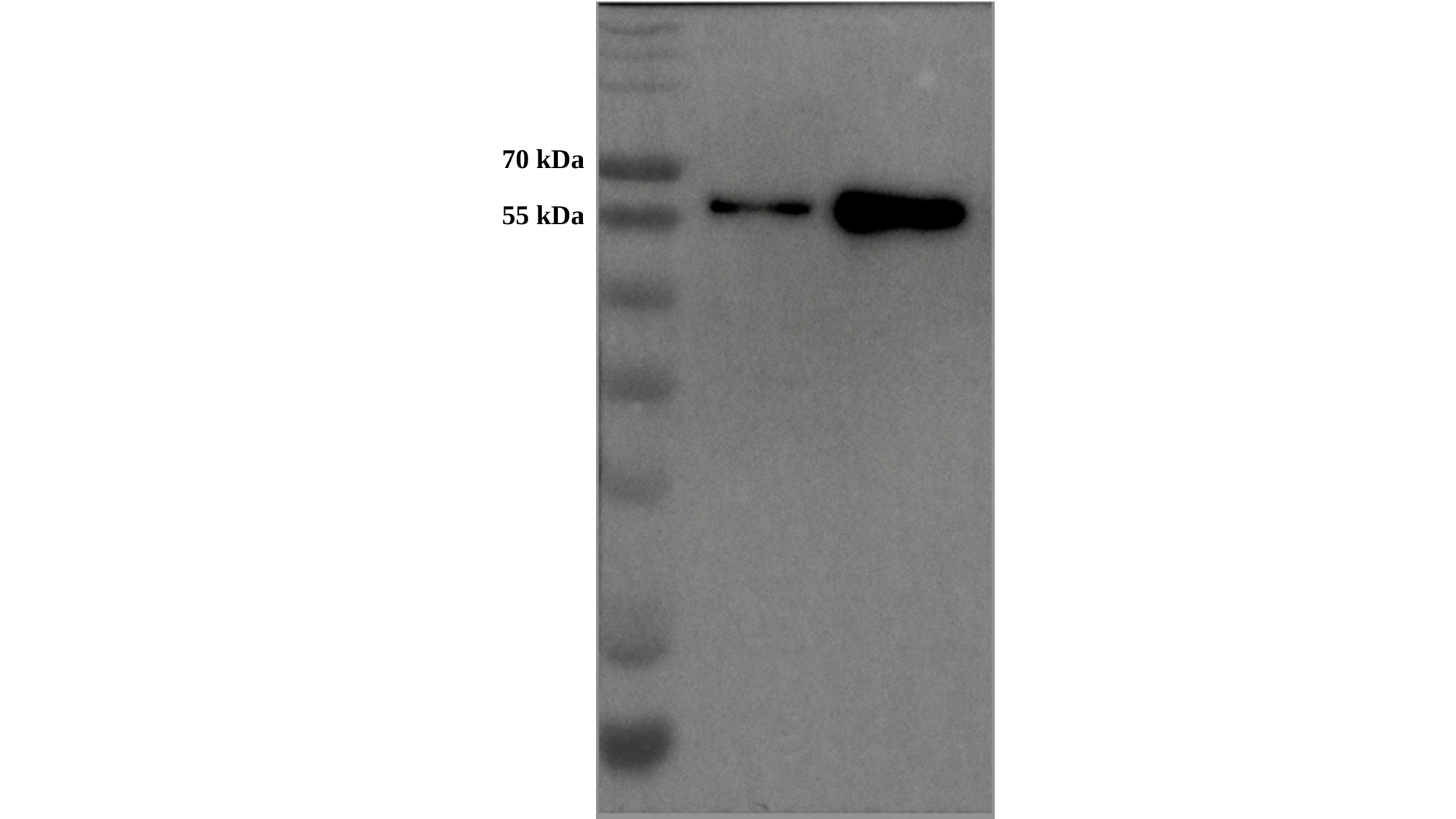

70 kDa
55 kDa

## Slide 6
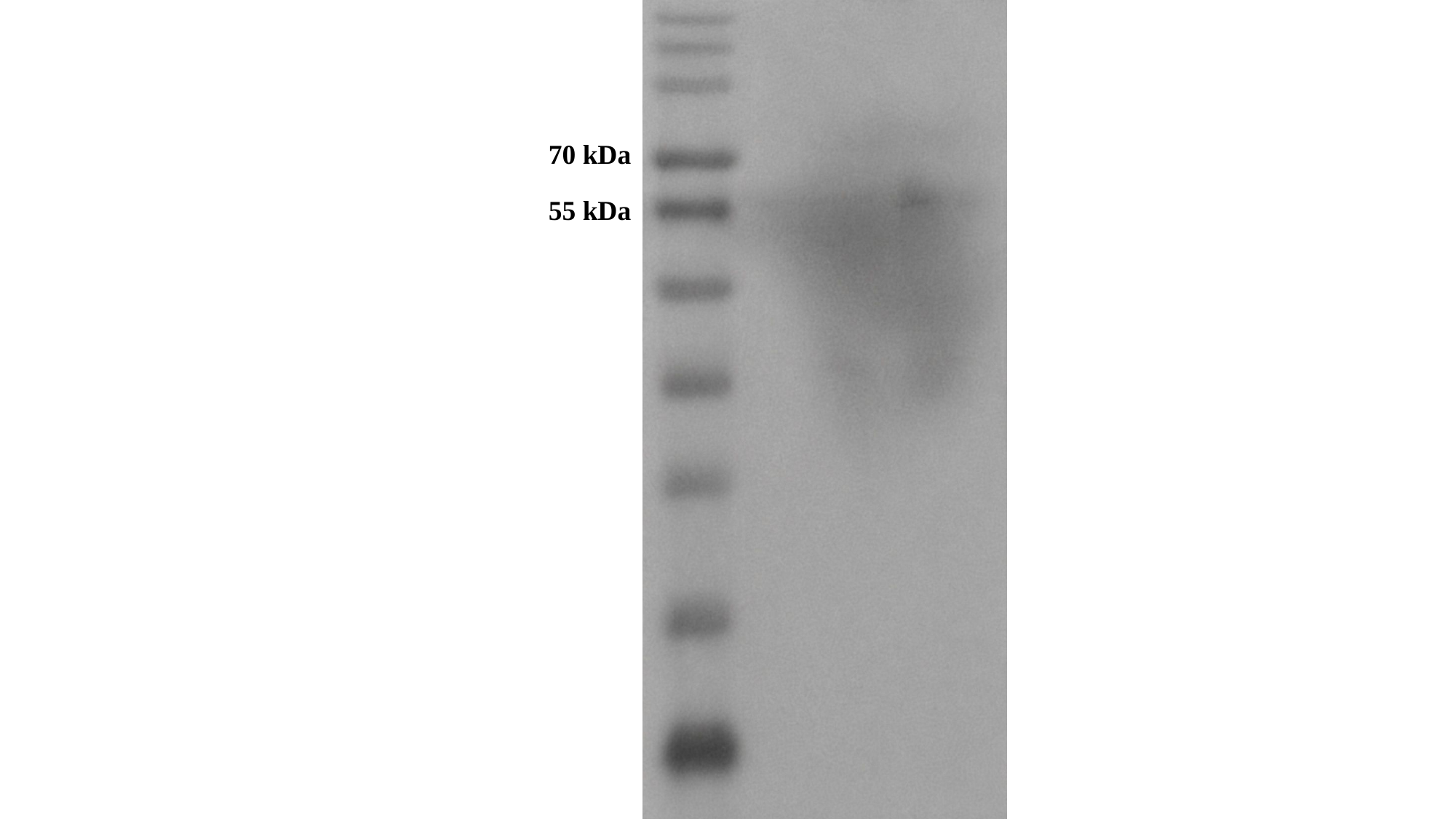

70 kDa
55 kDa

Supplement: Supplementary file 1 [file vaccines-14-00512-s001.zip › vaccines-4271232-supplementary.pptx]
